# Future projection of radiocesium flux to the ocean from the largest river impacted by Fukushima Daiichi Nuclear Power Plant

**DOI:** 10.1038/srep08408

**Published:** 2015-02-12

**Authors:** Mochamad Adhiraga Pratama, Minoru Yoneda, Yoko Shimada, Yasuto Matsui, Yosuke Yamashiki

**Affiliations:** 1 Environmental Risk Analysis Laboratory, Department of Environmental Engineering, Kyoto University; 2 Graduate School of Advanced Integrated Studies in Human Survivability, Kyoto University

## Abstract

Following the initial fall out from Fukushima Dai-ichi Nuclear Power Plant (FDNPP), a significant amount of radiocesium has been discharged from Abukuma River into the Pacific Ocean. This study attempted to numerically simulate the flux of radiocesium into Abukuma River by developing the multiple compartment model which incorporate the transport process of the radionuclide from the ground surface of the catchment area into the river, a process called wash off. The results from the model show that the sub-basins with a high percentage of forest area release the radionuclides at lower rate compared to the other sub-basins. In addition the results show that the model could predict the seasonal pattern of the observed data. Despite the overestimation observed between the modeled data and the observed data, the values of R^2^ obtained from ^137^Cs and ^134^Cs of 0.98 and 0.97 respectively demonstrate the accuracy of the model. Prediction of the discharge from the basin area for 100 years after the accident shows that, the flux of radiocesium into the Pacific Ocean is still relatively high with an order of magnitude of 10^9^ bq.month^−1^ while the total accumulation of the discharge is 111 TBq for ^137^Cs and 44 TBq for ^134^Cs.

An earthquake of magnitude 9.0 on the Richter scale occurred in Japan in March 2011 followed by 5 to 8 meter high tsunami waves hitting the east coast of Tohoku including the Fukushima Dai-ichi Nuclear Power Plant (FDNPP). The disaster caused a reactor meltdown which led to an explosion and the emission of radioactive substances to the environment. Based on surveys made by the Emergency Operation Center, Ministry of Education, Culture, Sports, Science and Technology, radiocesium was deposited in Fukushima Prefecture in the range of 10^4^–1.1 × 10^7^ Bq.m^−2^. The intrusion of the radionuclide in the environment led to the dispersion and migration process from one environmental compartment to others. This is confirmed by the detection of radiocesium and radiostrontium from samples of rivers (river water and riverbed sediment) in Fukushima Prefecture^1^. Moreover, the presence of radiocesium in Abukuma River had been reported by the Japanese Nuclear Regulation Authority. ^134^Cs and ^137^Cs activity concentrations were found to be very low in river water with measurement of, 0–2.77 Bq.l^−1^ for ^134^Cs and 0–4.77 Bq.l^−1^ for ^137^Cs, however, very high activities of ^134^Cs and ^137^Cs concentration were found in bottom sediment with measurement of, 0–14,000 Bq.kg^−1^ for ^134^Cs and 0–16,000 Bq.kg^−1^ for ^137^Cs^2^. The previous study shows that the presence of radiocesium is accumulated and concentrated in the top layer of the bottom sediment bed which suggests that the presence of the radionuclide originated from the FDNPP accident^3^.

According to the previous study conducted by Yamashiki et al.^4^, about 5.74 Tbq of ^137^Cs and 4.74 Tbq of ^134^Cs were discharged into the Pacific Ocean from Abukuma River during August 2011 to May 2012^4^. Moreover, about 85–92% of radiocesium transport is in particulate form which indicates that the transport of ^137^Cs in solid form plays an important role. In addition, the amount of radiocesium discharge previously mentioned is only 1.13% of the total radionuclides deposited in the basin area suggesting that about 98% of the radiocesium remains in the surface of the catchment area available for long term discharge. This condition requires the simulation of the transport process of the radionuclide from the basin area into the river, in order to allow further discharge to be predicted.

Models of radionuclide transport have been developed in many studies based on nuclear weapon tests^5^,^6^,^7^,^8^ and the Chernobyl nuclear power plant accident^9^,^10^,^11^,^12^,^13^,^14^,^15^,^16^,^17^ with various approaches and complexity levels. According to those previous studies, in general, soon after being deposited onto the ground surface, some fraction of the radionuclide migrates into the water body (rapid wash off) while the remaining part is stored in the catchment area for long-term migration (long term wash off)^6^. The wash off process is divided into two types based on the carrying media. When the radionuclide is transported by soluble media, the process is referred to as “liquid wash off” and when the migration occurs in particulate form, the process is called “solid wash off”^18^. Quantification of liquid wash off and solid wash off is governed by the wash off coefficients, K_l_ and K_s_ respectively. The rate of washed off radionuclide is represented by a transfer function which characterizes wash off as a function of time after a unit pulse of contamination by atmospheric deposition. The value was derived based on data from rivers in Europe. It is yet to be determined if the value is applicable to other locations, for example in Japan after the Fukushima accident.

The present study aims to develop a model for migration of radiocesium from the catchment area to the river which incorporates both the liquid and solid wash off. The Abukuma River is the second longest river in the Tōhoku region of Japan and the 6th longest river in Japan. The 234 km long river consists of 29 sub-basins flowing south to north (Figure 1). Two stations in the main channel of Abukuma River (Fushiguro and Iwanuma) were used for monitoring the flux of radiocesium in the previous study^4^. Fushiguro is located in the middle of the basin, whereas Iwanuma is located close to the mouth of Abukuma River in Sendai Bay. Figure 1 also shows the amount of radiocesium deposited in the basin area. It shows that the deposition was in the range of 0–2.25 × 10^6^ Bq.m^−2^. The middle part of the basin area received the highest deposition since it is the closest area to FDNPP. The results of the model were reviewed and compared with the field observation data obtained from Iwanuma Stations. In addition, the future flux of radiocesium from Abukuma River into the Pacific Ocean was estimated in this study.

## Results

**Figure 2:**
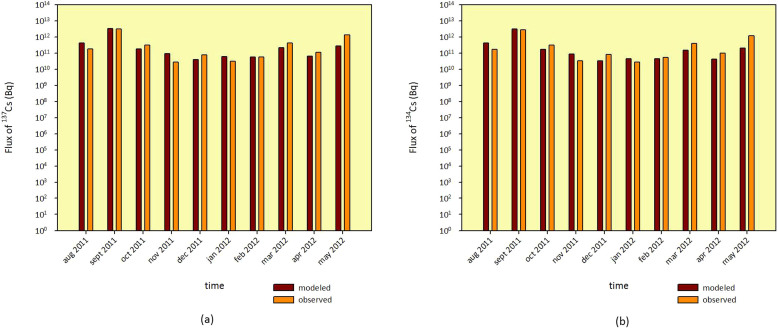
The comparison of modeled data and observed data for ^137^Cs (a) and ^134^Cs (b).

For testing the accuracy of the model, the estimated data resulting from the model were compared to the observed data as presented in Figure 2a and 2b. An acceptable value of R^2^ and Nash efficiency coefficient were obtained. The value of R^2^ of 0.86 and the Nash efficiency coefficient value of 0.85 were achieved for both ^137^Cs and ^134^Cs indicating the model could accurately predict the seasonal variation and the amount of the flux. Based on the estimation, the flux of radiocesium was high in September 2011 in which about 6.5 × 10^12^ Bq of radiocesium was discharged into the Pacific Ocean through Sendai Bay. On the other hand, the flux of radiocesium reached the minimum level during winter season from December to February 2011. Moreover the discharge gradually increased from March to May of 2012 as the rainfall rate increased.

The comparison shows that the model could explain the seasonal variation of the observed data. The estimated flux of radiocesium reaches the peak during high rainfall months. In September of 2011, a heavy storm occurred during the 19^th^–23^rd^ during which 157 mm of rainfall was precipitated. This phenomenon was the major event of radiocesium transport into Abukuma River. It was estimated that during August of 2011 to May of 2012, about 71% of the discharge occurred during September. The huge amount of radiocesium discharge during September 2011 was observed from the model. The escalation of the discharge was dramatically increased compared to the previous month.

**Figure 3:**
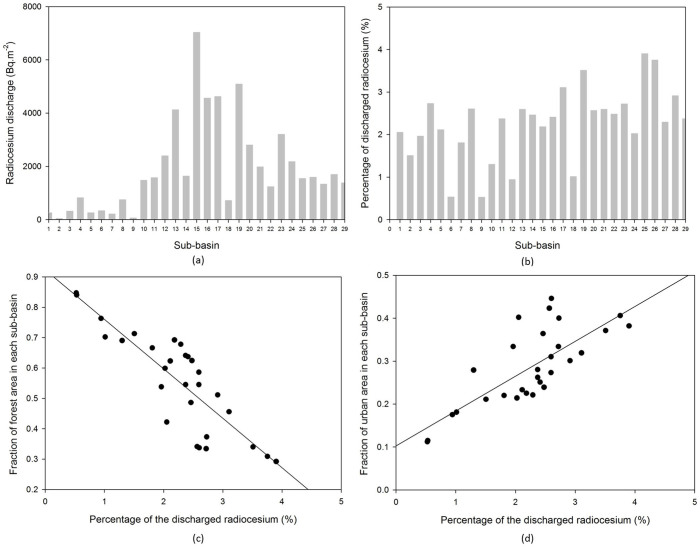
The discharge of radiocesium from each sub-basin during August 2011–May 2012 (a) and the percentage of released radiocesium of total estimated deposition for each sub-basin (b). The correlation of the percentage of released radiocesium of total estimated deposition for each sub-basin with the percentage of forest area (c) and urban area (d).

The discharge of radiocesium during August 2011 to May 2012 from each sub-basin is presented in Figure 3a. The average amount of radiocesium discharge for the whole basin in that period was 41,904 Bq.m^−2^. Sub-basin 15 discharges the highest amount of radionuclides into Abukuma River, and it was estimated that the sub-basin released about 7,033 Bq.m^−2^. On the other hand, the lowest discharge was found from Sub-basin 2 where the amount of radiocesium released in the same period of time was about 38 Bq.m^−2^.

The amount of fall-out is strongly correlated with the amount of the radiocesium that is discharged from each sub-basin. The middle part of the basin (Sub-basin 14,15 and 16) which received the highest amount of the fall out discharges the highest amount of radiocesium. On the other hand, since the downstream part of the river basin received a lower amount of fall out, the discharge is relatively a lower than the middle part.

The amount of radiocesium discharged from each sub-basin as a percentage of the total initial fall-out is shown in Figure 3b. The percentage is in the range of 1–4% with an average of 2% for the whole basin. Sub-basin 19, 25 and 26 have the highest percentage (7%) whereas sub-basin 6, 9 and 18 are the lowest (3%). Figure 3c and 3d show the correlation between the percentage of the discharged radiocesium and the percentage of forest and urban area of the basin respectively. The R^2^ value between percentage of the discharged radiocesium and the percentage of forest area of −0.84 show that the sub-basins with high percentage of forest area release the radionuclide at a lower rate. On the other hand, with an R^2^ value of 0.76, sub-basins with a high percentage of urban area release radionuclide at a higher rate.

It is generally known that surface runoff and sediment yield in the forest area is relatively low compared to urban or agriculture areas. Forest has a high capacity to store the precipitated water from rainfall. Thus, when rainfall occurs, more water is infiltrated into the ground than flowing on the surface. As a result, the amount of liquid wash off is low. Moreover, the tree roots which penetrates the soil ground strengthens the soil layer and reduces the detachment process of soil particles. Consequently, the solid wash off from the forest area would be minimized. It was confirmed that the contamination in the bottom sediment of the upper part of the basin is lower than in the soil nearby while in the coastal area, the contamination is higher^19^. This shows that the rate of radiocesium migration is lower in the upper part of the catchment area in which the land cover is dominated by forest.

**Figure 4:**
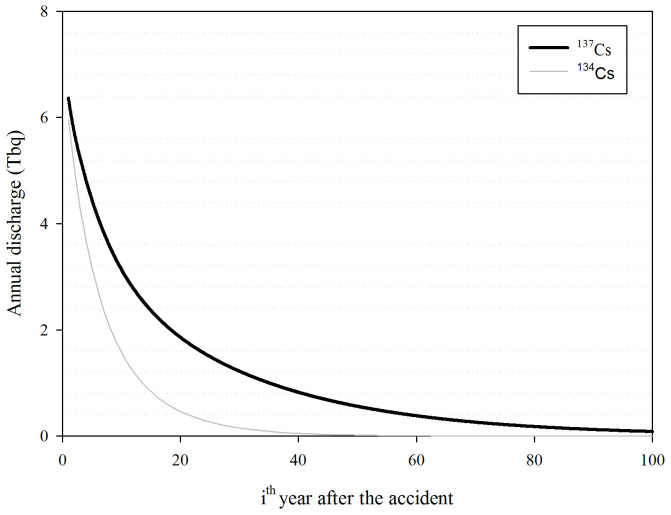
The forecast of radiocesium flux into the Pacific Ocean for 25 years after the accident.

**Figure 5:**
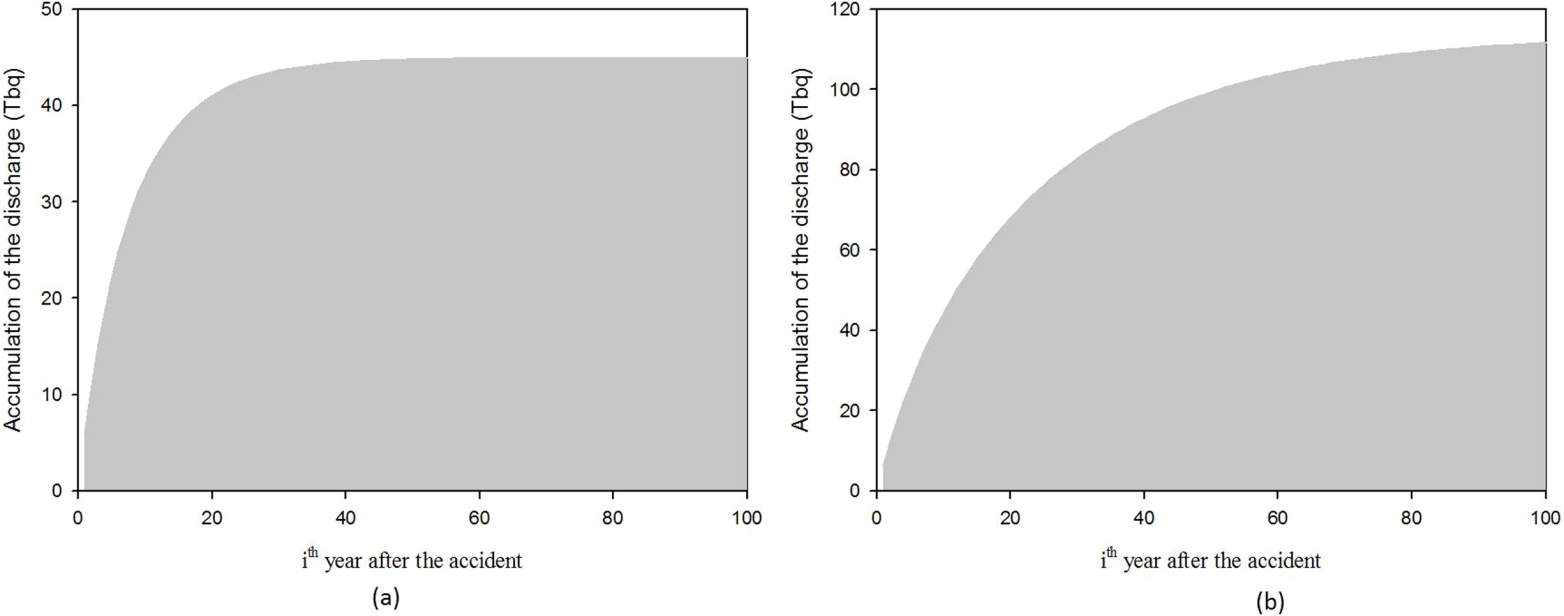
The accumulation of ^134^Cs (a) and ^137^Cs (b) flux into Pacific Ocean for 100 years after the accident.

In order to predict the further discharge of radiocesium, the forecast of the radiocesium flux from Abukuma River into the Pacific Ocean for 100 years after the accident is shown in Figure 4. The weathering effect was determined based on meteorological data collected from 1994 to 2012 provided by Automated Meteorological Data Acquisition System (AMeDAS) developed by Japan Meteorological Agency (JMA). The figure shows in 2111 (100 years after the accident) the flux is still high at 5.62 × 10^9^ bq.month^−1^. The other estimation was a calculation the accumulation of radiocesium discharge into the Pacific Ocean for 100 years (Figure 5a and 5b). The estimation shows that 111 TBq of ^137^Cs and 44 TBq of ^134^Cs are discharged during that period.

**Figure 6:**
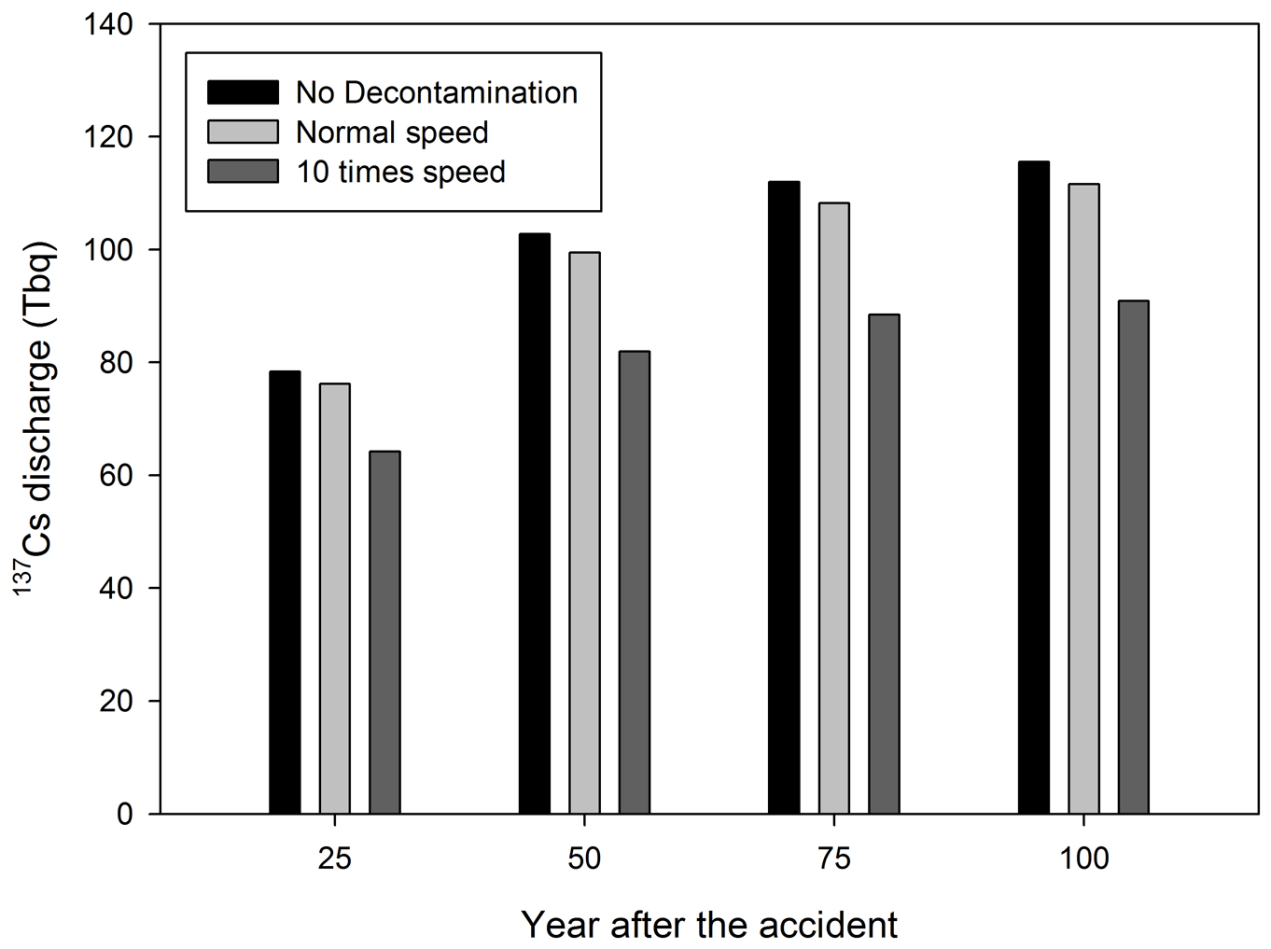
The effect of decontamination activities to the discharge of radiocesium into the Pacific Ocean for 100 years.

The effect of decontamination activities is presented in Figure 6. It can be seen that without decontamination, the total discharged ^137^Cs into the Pacific Ocean after 100 years is 114 Tbq. By considering the decontamination activities with normal speed, the discharge after 100 years is 111 Tbq. A significant difference was obtained by increasing the decontamination by 10 times in which the discharge becomes 90 Tbq after 100 years. The normal speed of the decontamination activities suggested by JAEA might reduce a significant amount of radiocesium dose in the urban area. However, from the point of view of the amount of radiocesium in the basin scale, the suggested speed does not decrease the radiocesium flux significantly. As mentioned before, the reduction of the flux could be seen if the speed of the decontamination is at least 100 times the suggested speed. However, it should be noted that increasing the speed requires a higher cost to carry out decontamination.

## Discussion

Several uncertainties leading to the difference between modeled and observed data were observed. One of the factors leading to uncertainty of the result is that the size of the suspended particles has not been considered. Smaller size particles tend to adsorb more radiocesium^20^. The lack of consideration of the distribution of suspended particle size is the causes of the difference between observed and modeled data.

The model shows that the result could explain the seasonal variation of flux of radiocesium in Abukuma River. With R^2^ value of 0.86 and the Nash efficiency coefficient value of 0.85, it might be concluded that the model predicts well. Despite the good value, there was overestimation between the modeled and the observed data. This condition shows that the wash off coefficient obtained from studies based on nuclear weapon test and post Chernobyl should be evaluated for application in Japan. This could be due to the difference of soil type in the forest or material used in the ground surface of the urban area.

The future prediction of the discharge estimates that 111 TBq of ^137^Cs and 44 TBq of ^134^Cs are released into the Pacific Ocean during 2011–2111. This condition shows that prevention of the radioactive substance leakage from the FDNPP is insufficient since river as the media of radioactive substance transport is also significant source. While the discharge from Abukuma River is equal to 30% of FDNPP direct discharge and also considering the discharge of the radioactive substance from the other major rivers in Fukushima Prefecture (Arakawa, Naka, Agano, Tadami), the sum of the discharge from these rivers could be as large as the leakage from FDNPP. Although this statement needs confirmation, it is sufficient to warrant concern from the authorities of rivers as a major source of radiocesium flux into the Pacific ocean.

Since a huge amount of radiocesium is discharged into the Pacific Ocean, it is important to characterize the plume of radiocesium in the estuary of Abukuma River for further analysis of the distribution of the radionuclide in the coastal area. This is deemed necessary since the presence of radiocesium in the coastal area could be transferred and accumulated into the biotic component of the environment. Furthermore this prediction of radiocesium discharge could be used for predicting the transfer of radiocesium from abiotic component (dissolved in river or sea water and solid form attached in suspended solid) into biotic component (Fish, benthic, mollusk). Although the numerical lumped model used in this study gives a good R^2^ value, it could be improved by application of a numerical distributed model. Using a 250 × 250 m or 500 × 500 m grid, the transport of the radionuclide will be calculated in more detail and it is expected that the overestimation of the modeled data that occurred in this study could be minimized.

## Methods

### The Model

The model developed in this study consists of two sub-model, namely “BASIN” and “RIVER”. Based on the land cover type on the surface of the catchment area, sub model BASIN is divided into Forest, Urban and Agriculture area. The following sections describe both sub-models in detail.

### Sub-Model “BASIN”

This sub-model uses compartment system to describe the process considered in this study. Each land cover type consists of the different compartments, however, each compartment is governed by the following equation:
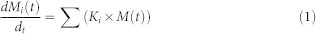
Where M_i_(t) is the amount of radionuclide in compartment i (bq) and K_i_ is the transfer rate of the radionuclide into another compartment.

### Forest Area

**Figure 7:**
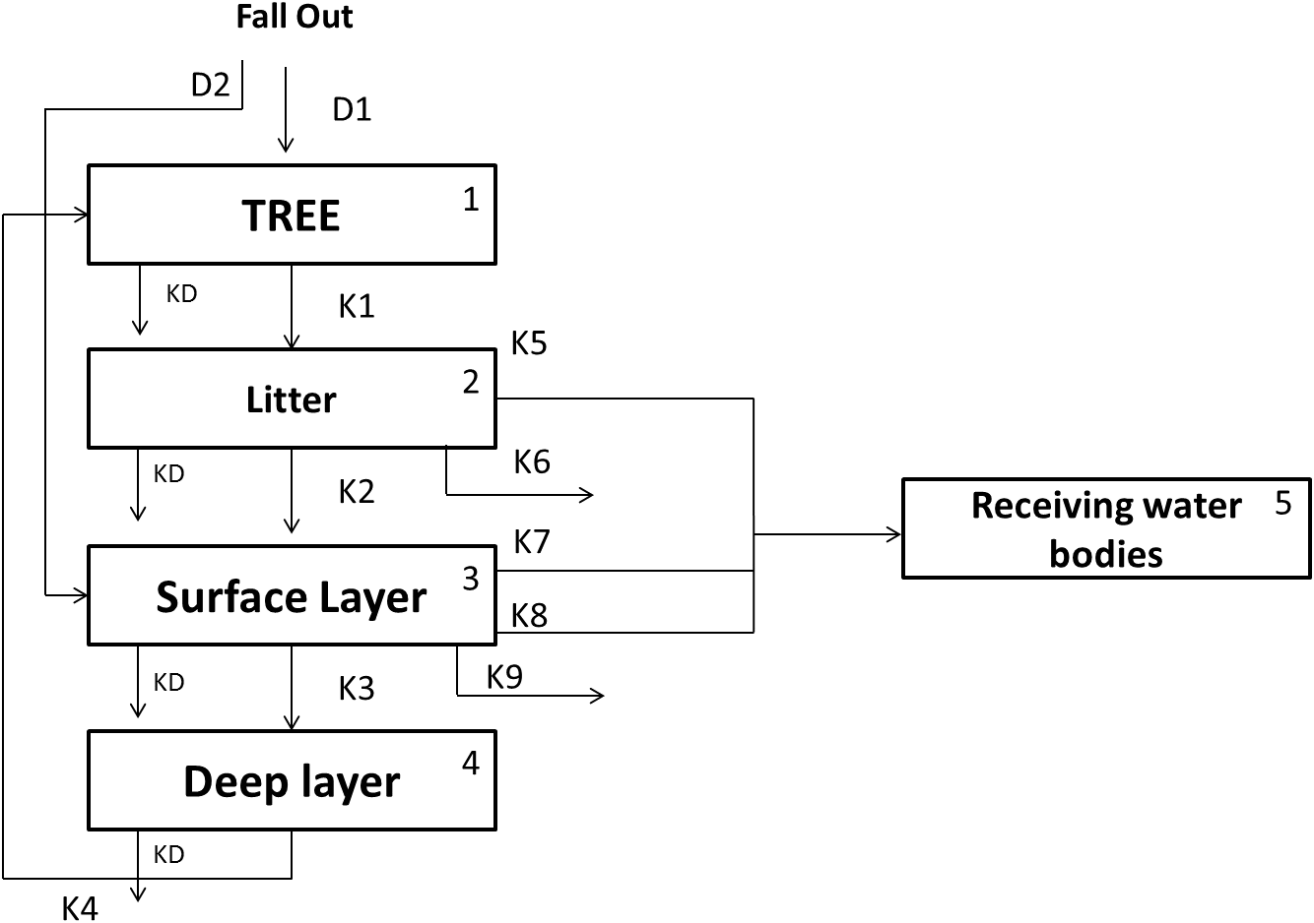
The network system of forest area in Sub Model “BASIN”.

The network system in forest area developed in this model is presented in figure 7. The system consists of 5 compartments namely “Tree”, “Litter”, “Surface Layer”, “Deep Layer” and the “receiving water bodies”.

The detailed process in this network is described as follows:

- For each compartment, the amount of the radionuclide is naturally decreased by decay process which is represented by a transfer rate called Kd.
- The radionuclide in the atmosphere is deposited onto the ground surface of the forest area. Some part of it is intercepted by the tree surface(D_1_) and the other is directly on deposited in the soil surface (D_2_).
- Following the lifecycle of a tree, the radionuclide attached to the surface of leaves falls into the ground surface (K_1_) with a specific value of transfer rate during autumn season, creating a new compartment called “Litter”. The governing equation of compartment 1 is presented as follows:
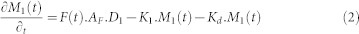
Where F(t) is the initial radionuclide deposition (bq.m^−2^) and A_F_ is the total area of the forest area (m^2^). The value K_1_ is not 0 only for autumn season (day 270–330).
- Several process affecting the dynamic of the radionuclide in the litter compartment. First, the migration of the radionuclide from the litter compartment to the surface layer compartment (K_2_) beneath. The radionuclide is also washed off by the run off surface at a specific rate (K_5_) flowing into the receiving water bodies. Decontamination activities carried out by people reduces the amount of radionuclide in this compartment driven by a rate represented in K_6_. The governing equation of compartment 2 is presented as follows:
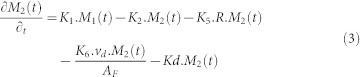
Where R is the surface run-off (mm) and v_d_ is the decontamination speed (m^2^).
- Processes occurring in the surface layer compartment are almost similar to those of the litter compartment which are Migration into deep layer (K_3_), Wash off process (K_7_) and Decontamination rate (K_9_). However, the migration of soil particles due to erosion process occurs in this compartment, therefore solid wash off process is considered (K_8_).The governing equation of compartment 3 is presented as follows:
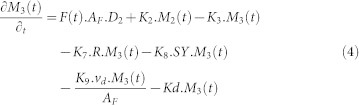
Where, SY is the sediment yield (g.m^−2^).
- In the deeper layer, the radionuclide is taken up by trees through the penetration of their roots (K_4_). The governing equation of this compartment is written as follows:
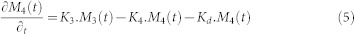

### Urban Area

**Figure 8:**
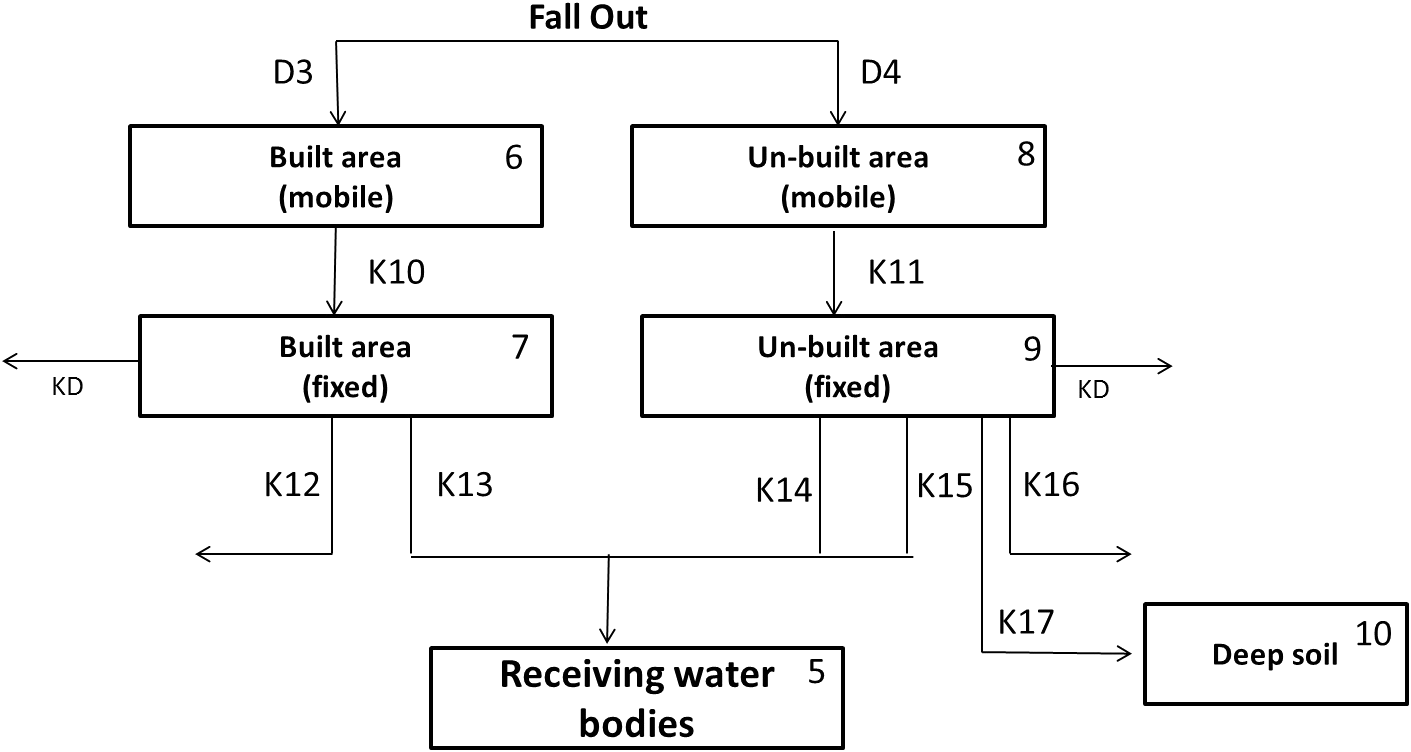
The network system of urban area in Sub Model “BASIN”.

Figure 8 describes the network system representing the fate of radionuclides in the urban area. The system consists of 6 compartments including built area and un-built area, deeper soil of un-built area and the receiving water bodies. The detailed process in this network is described as follows:

- Similar to the forest area, the amount of radionuclide in each compartment in the urban area is addressed by the natural decay process driven by K_D_.
- The radionuclide is deposited in two types of urban area, some fraction of the radionuclide falls onto the surface of built area (D_3_) and the other onto the un-built area (D_4_).
- On the surface of urban area, the radionuclide is initially presents in mobile form (Compartment 7 and 9). About 60–80% of mobile form will be swept by the first heavy rainfall (>1 mm). The remaining part is fixated into the fixed form (Compartment 8 and 10). The governing equation of compartment 7 and 9 is written as follows:
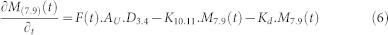
Where, A_U_ is the total area of urban area in the catchment (m^2^).
- The radionuclide in fixed form in the built area (Compartment 7) is subjected to the removal process by decontamination activities (K_12_) and the liquid wash off process (K_13_) as written in the following equation.
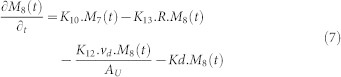
- In compartment 9, besides removal due to the decontamination activities (K_16_) and the liquid wash off process (K_14_), the radionuclide is migrated into the receiving water bodies through solid wash off (K_15_) and also migrated into the deeper soil layer (K_17_). The governing equation in compartment 9 is written as follows:
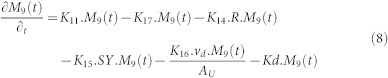
- In the deeper layer, the radionuclide will be accumulated according to the value of K_17_ also subjected to natural decay contamination as written in the following equation.
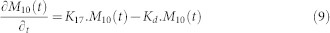

### Agriculture Area

**Figure 9:**
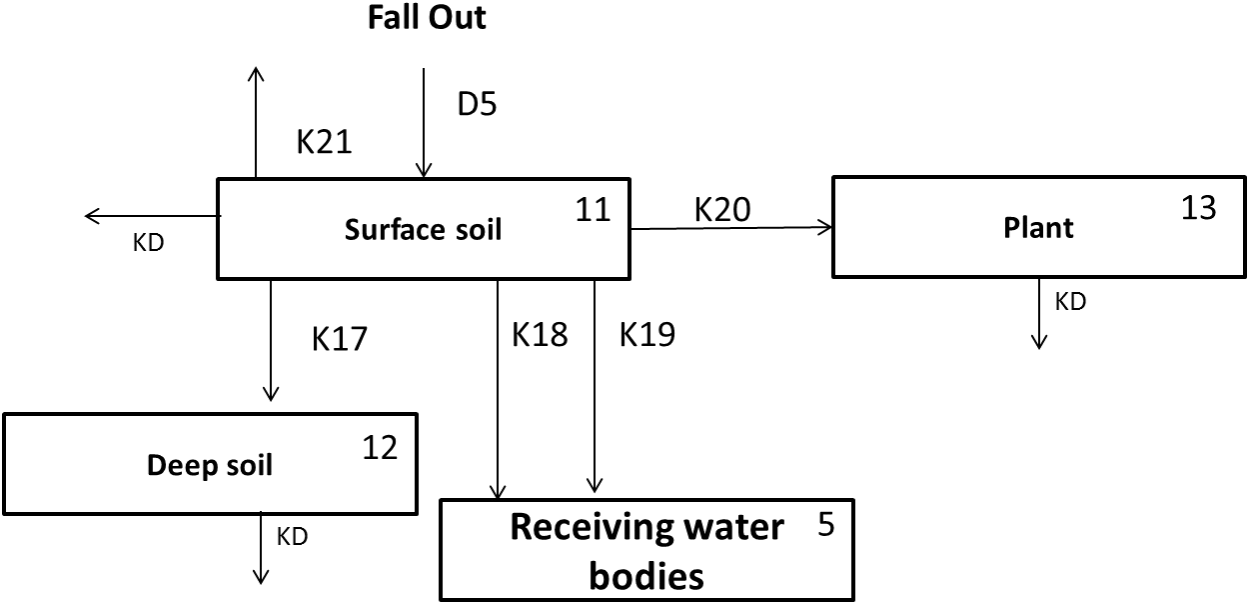
The network system of agriculture area in Sub Model “BASIN”.

The process in agriculture area is simpler than in the previous area as presented in the figure 9. The system consists of 4 compartments including the surface layer, deeper layer, the cultivated plant and the receiving water bodies. The detail process in this network is described as follows:

- The amount of radionuclide in each compartment in urban area is subjected by the natural decay process driven by K_D_.
- The radionuclide in the atmosphere falls onto the surface layer of the agriculture area (D_5_). The dynamic of radionuclide in this compartment is affected by: migration to deeper soil (K_17_), liquid wash off (K_18_), solid wash off (K_19_), uptake to cultivated plant (K_20_) and the decontamination activity (K_21_). The governing equation is written as follows:
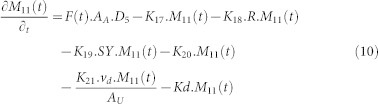
A_U_ represents the total area of agriculture area in the catchment area.
- The Compartment 13 represents the cultivated plant compartment. The transfer rate (K_20_) has a value greater than zero only during planting to harvesting period, otherwise the process is neglected since there are no plants that are cultivated. The equation for this compartment is written as follows:
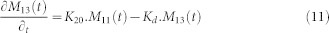

### Parameter values

The values for each parameter used in this sub-model collected from various studies are presented in Table 1.

### Sub-Model “RIVER”

Flux of radionuclide both from the liquid wash off process and the solid wash off process enters the river environment through the outlet of each sub-basin. In the river environment, radionuclide which is initially present in dissolved form, is adsorbed into suspended solid form. The sorption process depends on the value of the partition coefficient (Kd). Then, based on the settling velocity, radionuclide is either transported by the river stream in the downstream direction before being deposited onto the sediment bed or is deposited directly onto the sediment bed. Physical and chemical characteristics of soil particles, river water, suspended solids and sediment particles play an important role for determining the significance of each process.

Based on the location of each sub-basin outlet, this sub-model divides Abukuma River into nine sections. The sedimentation process of the suspended particles plays an important role since most of the radionuclides is transported in the solid form.
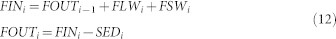
Where, FIN_i_ is the flux of the radionuclide entering Section i (bq.day^−1^). FOUT_i-1_ represents the flux of radionuclide exiting the previous Section (bq.day^−1^). FLW_i_ and FSW_i_ represent the flux of radionuclide from the sub-basins which have their outlet located in Section i (bq.day^−1^). FOUT_i_ is the flux of radionuclide exiting Section i (bq.day^−1^) and SED_i_ represents the amount of radionuclide removed from the stream due to the sedimentation process.

Each section is characterized by a specific value of the sedimentation rate which is obtained from the following equation proposed by Ref. 24:
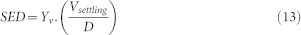
V_settling_ is the settling velocity of the suspended particulate and D is the depth of the water column in the section. Yv is the dimensionless moderator which expresses how river water velocity V_river_ (Q/A) influences sedimentation. Yv is defined by:
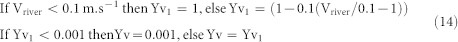
In order to calculate the sedimentation rate, we collected the flow rate and the water level data for each section from the last two years provided by the Japanese Ministry of Land, Infrastructure and Transport. The summary of the sedimentation rate, flow rate and water level for each section is shown in Table 2. The flow rate of Abukuma River varies from 21 m^3^.s^−1^ in the first station, Sukagawa, to 164 m^3^.s^−1^ in the ninth station, Iwanuma. The highest sedimentation rate was found in the fourth station, Nihonmatsu, at around 0.32 day^−1^.

Moreover, the significant process reducing the flux of radionuclides in the river system is the deposition of solid form in the intermediate storage. In the Abukuma river system, there are at least four big reservoirs which are presented in table 3. The settling velocity for each reservoir uses general value proposed by Monte, which is 1.10^−6^ (m.s^−1^)^12^.

### Utilization of SWAT Model

In this sub-model, the amount of surface runoff and suspended particle yield are the important components since the amount of wash off depends on these components. Soil Water Assessment Tool (SWAT) which is integrated into the ArcMAP10.0 (ESRI) interface was used to determine the amount of surface runoff and sediment yield generated in the sub-basins. A digital Elevation Model (DEM) of Fukushima and Miyagi Prefecture were collected for delineating the perimeter of Abukuma river basin area. Hydrologic response unit (HRU) was then defined by collecting land use, soil type and slope data. Landsat8 data of both prefectures were collected and by using the image classification tool in ArcMAP 10.0, a land-use map of the basin area was obtained. Together with soil type data obtained from the world soil map and the land slope derived from DEM, HRU was defined. Before running the model, Abukuma River basin weather data consisting of daily rainfall, wind velocity, humidity and solar radiation obtained from Automated Meteorological Data Acquisition System (AMeDAS) were collected from 1990 to 2012 to calculate the weathering effect. The SWAT model was run at a daily time step to obtain the daily amount of surface runoff (mm) and suspended particulate yield (metric ton.ha-1) for each sub-basin.

### The Effect of Decontamination Activities

The Japanese government has been carrying out the decontamination activities since 2011. According to the document released by the Japanese Atomic Energy Agency, there are specific decontamination method for specific land use type including the removal rate and the decontamination speed as presented in Table 4. In this study, three scenarios were developed to estimate the discharge of radiocesium over 100 years without decontamination, with normal decontamination and with decontamination increasing the speed by 10 times.

## Author Contributions

M.A. and Y.Y. wrote main components of the article. M.Y. designed the model and gave advice. Y.S. made detailed work on the model. Y.M. gave instruction on the model. M.A. made overall calculation and finalization of the work. Y.Y. designed the structure of the article, gave observation results, and gave advice.

## Figures and Tables

**Figure 1:**
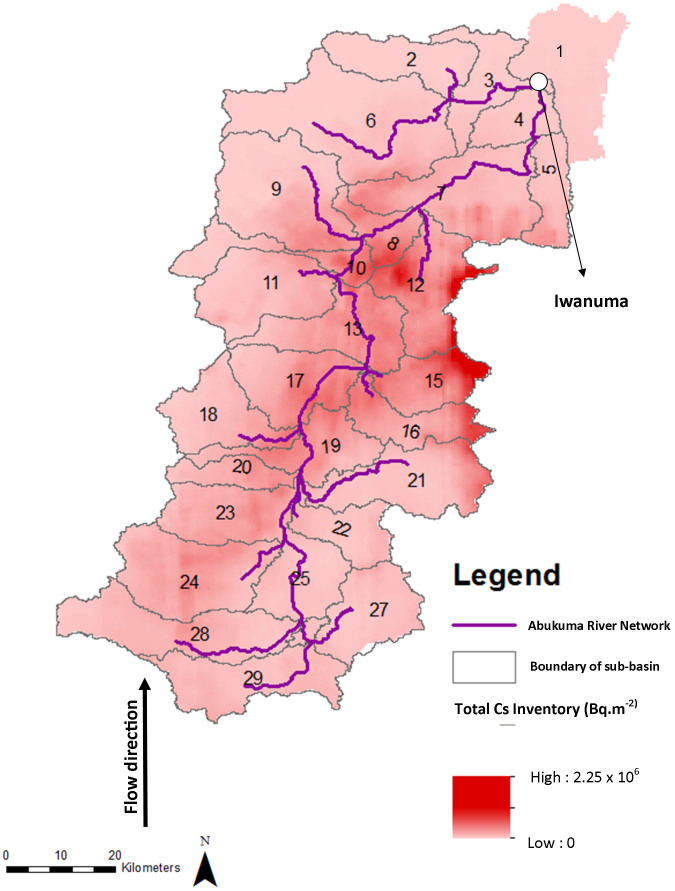
Abukuma River Basin, sub-basin perimeter, deposition of radiocesium and the location of two monitoring stations. (Map was modified based on the figure used in previous study^4^ by using ArcMap 10.1 (ESRI), GIS data were obtained from Japan Map Center).

**Table 1:** The values for each parameter used in sub-model “BASIN” collected from various studies

No	Parameter	Definition	Value	Unit	Source
1	D1	fraction of interception by canopy	0.8	-	
2	D2	fraction of deposition fall on surface of soil surface	0.2	-	
3	D3	fraction of deposition fall on built area	0.8	-	
4	D4	fraction of deposition fall on unbuilt area	0.2	-	
5	D5	fraction of deposition fall on agriculture	0.15	-	
6	K1	migration rate of radionuclide due to leave fall	0.009–0.03	/day	26
7	K2	migration rate of radionuclide from litter to surface soil	0.006–0.6	/day	26
8	K3	migration rate of radionuclide from surface to deep soil	6.6e − 6–8.0e − 5	/day	21,25
9	K4	upatake rate of radionuclide from deep soil to tree	0.006–0.6	/day	26
10	K5	liquid wash off rate from litter compartment	1.9e − 6–1.2e − 4	/mm	9,18
11	K6	decontamination rate of litter compartment	90	%	27
12	K7	liquid wash off rate from surface soil	1.9e − 6–1.2e − 4	/mm	9,18
13	K8	solid wash off rate from surface soil	8.2e − 5–6.7e − 4	m^2^/g	9,18,28
14	K9	decontamination rate of surfacesoil	80	%	27
15	K10	fixation rate of radionuclide in built area	0.1–1	/day	21,25
16	K11	fixation rate of radionuclide in unbuilt area	0.1–1	/day	21,25
17	K12	decontamination rate in built area	70	%	27
18	K13	liquid wash off rate in built area	5e − 4–3e − 3	/mm	21,25
19	K14	liquid wash off rate in unbuilt area	1.9e − 6–1.2e − 4	/mm	21,25
20	K15	solid wash off rate in unbuilt area	1.6e − 5–6.7e − 4	m^2^/g	9,18
21	K16	decontamination rate in unbuilt area	70	%	27
22	K17	migration of radionuclide in unbuilt area to deep soil	6.6e − 6–8.0e − 5	/day	21,25
23	K18	liquid wash off rate in surface soil of farmland	1.9e − 6–1.2e − 4	/mm	9,18
24	K19	solid wash off rate in surface soil of farmland	1e − 5–6.7e − 4	m^2^/g	9,18,28
25	K20	uptake rate from of plant	0.00017	/day	29
26	K21	decontamination rate of farmland	70	%	27
27	KD	half-life of ^137^cs	30.1	year	-
		half-life of ^134^cs	8	year	

**Table 2:** The location of sub-basin outlet, water level and flow rate of 9 sections in Abukuma River

			Water Level (m)		Flow Rate (m^3^.s^−1^)	
No	station	Sub-basin	min	max	min	max
1	Sukagawa	26,27,28,29	1.17	6.25	7.23	419.47
2	Miyota	24,25	0.13	4.22	7.37	1212.12
3	Motomiya	18,19,20,21,22,23	1.5	7.63	21.93	1878.59
4	Nihonmatsu	14,15,16,17	2.69	9.18	23.42	2211.04
5	Kuroiwa	11,13	0.97	5.51	19.32	2511.81
6	Fushiguro	8,12	1.56	3.4	33.91	3348.67
7	Fukushima	9,10	1.18	3.89	42.6	2679.94
8	Marumori	5,7	12.3	15.5	48.7	1236.57
9	Iwanuma	1,2,3,4,6	14.63	17.73	91.54	1553.04

**Table 3:** Intermediate storage of Abukuma River system

Reservoir Name	Level (m)	Sub-basin	Sedimentation Rate (/month)
Miharu	65	20	0.0393
Surikamigawa	105	8	0.0246
Sichikasuku	90	5	0.0288
Horai	21.5	>14	0.12

**Table 4:** Decontamination method suggested by JAEA^27^

No	Compartment	Method	Removal rate (%)	Decontamination speed (m^2^/day)
1	Litter	Removal of leaf litter and humus layers	5–90	510
2	Surface soil of forest	Removal of humus layers and topsoil	20–80	220
3	Built area	Ultra-high-pressure water-based washing (150 Mpa or higher)	70	300
4	Unbuilt area	Thin-layer soil stripping equipment (hammer knife)	70	700
5	Agriculture surfacesoil	Thin-layer soil stripping equipment (hammer knife)	70	700
